# Differential neutrophil activation in viral infections: Enhanced TLR‐7/8‐mediated CXCL8 release in asthma

**DOI:** 10.1111/resp.12657

**Published:** 2015-10-18

**Authors:** Francesca S.M. Tang, David Van Ly, Kirsten Spann, Patrick C. Reading, Janette K. Burgess, Dominik Hartl, Katherine J. Baines, Brian G. Oliver

**Affiliations:** ^1^ Woolcock Institute of Medical ResearchThe University of SydneySydneyNew South WalesAustralia; ^2^ Discipline of Pharmacology, School of Medical Sciences, Faculty of MedicineThe University of SydneySydneyNew South WalesAustralia; ^3^ Genome Integrity GroupThe Children's Medical Research InstituteSydneyNew South WalesAustralia; ^4^ School of Biomedical Science, Faculty of HealthQueensland University of TechnologyBrisbaneQueenslandAustralia; ^5^ WHO Collaborating Centre for Reference and Research on InfluenzaPeter Doherty Institute for Infection and ImmunityMelbourneVictoriaAustralia; ^6^ Priority Research Centre for Asthma and Respiratory DiseaseThe University of NewcastleNewcastleNew South WalesAustralia; ^7^ School of Medical and Molecular BiosciencesUniversity of Technology SydneySydneyNew South WalesAustralia; ^8^ Department of Pediatrics IUniversity of TübingenTübingenGermany

**Keywords:** asthma, innate immune responses, neutrophils, respiratory viruses, rhinovirus

## Abstract

**Background and objective:**

Respiratory viral infections are a major cause of asthma exacerbations. Neutrophils accumulate in the airways and the mechanisms that link neutrophilic inflammation, viral infections and exacerbations are unclear. This study aims to investigate anti‐viral responses in neutrophils from patients with and without asthma and to investigate if neutrophils can be directly activated by respiratory viruses.

**Methods:**

Neutrophils from peripheral blood from asthmatic and non‐asthmatic individuals were isolated and stimulated with lipopolysaccharide (LPS) (1 μg/mL), f‐met‐leu‐phe (fMLP) (100 nM), imiquimod (3 μg/mL), R848 (1.5 μg/mL), poly I:C (10 μg/mL), RV16 (multiplicity of infection (MOI)1), respiratory syncytial virus (RSV) (MOI1) or influenza virus (MOI1). Cell‐free supernatants were collected after 1 h of neutrophil elastase (NE) and matrix metalloproteinase (MMP)‐9 release, or after 24 h for CXCL8 release.

**Results:**

LPS, fMLP, imiquimod and R848 stimulated the release of CXCL8, NE and MMP‐9 whereas poly I:C selectively induced CXCL8 release only. R848‐induced CXCL8 release was enhanced in neutrophils from asthmatics compared with non‐asthmatic cells (*P* < 0.01). RSV triggered the release of CXCL8 and NE from neutrophils, whereas RV16 or influenza had no effect.

**Conclusion:**

Neutrophils release CXCL8, NE and MMP‐9 in response to viral surrogates with R848‐induced CXCL8 release being specifically enhanced in asthmatic neutrophils. Toll‐like receptor (TLR7/8) dysregulation may play a role in neutrophilic inflammation in viral‐induced exacerbations.

## Abstract 2

We aimed to investigate and compare neutrophil responses to bacterial compounds and viral mimetics as well as compare responses between people with and without asthma. We also investigated neutrophil responses to live respiratory viruses. Here we provide a novel comprehensive comparison showing differential and specific activation in innate immune cells.

See Editorial, page 10

**Abbreviations**
ANOVA: analysis of variance
DMSO: dimethyl sulfoxide
ELISA: enzyme‐linked immunosorbent assay
FEV_1_: forced expiratory volume in 1 s
fMLP: f‐met‐leu‐phe
FVC: forced vital capacity
ICS: inhaled corticosteroid
IL: interleukin
LPS: lipopolysaccharide
MDA‐5: melanoma differentiation‐associated protein 5
MMP: matrix metalloproteinase
MOI: multiplicity of infection
NE: neutrophil elastase
PAMPs: pathogen‐associated molecular patterns
RSV: respiratory syncytial virus
RV: rhinovirus
TLR: toll‐like receptor

## Introduction

Neutrophilic asthma is a distinct asthmatic phenotype with poor therapeutic response and a lack of mechanistic insights.^1^ In these patients, neutrophil numbers and their secretory products, such as CXCL8, neutrophil elastase (NE) and matrix metalloproteinase (MMP)‐9, are elevated in the airways, particularly during exacerbation. These levels also correlate with greater severity of asthma symptoms.^2^, ^3^

Respiratory viral infections are a major cause of asthma exacerbations with rhinovirus (RV) accounting for up to 80% of all exacerbations.^4^ Airway neutrophilia can also occur transiently in response to viral infections.^5^ Neutrophils appear to be implicated both in asthma and during infections; however, the immune responses that regulate virus‐induced exacerbations and, in particular, neutrophilic inflammation remain poorly defined.

Neutrophils contain a range of receptors that recognize pathogen‐associated molecular patterns (PAMPs) present on bacteria, fungi and viruses. The majority of respiratory viruses are RNA viruses that can be detected by toll‐like receptors (TLRs) 3, 7 and 8. Studies have already described the expression of TLR7 and 8 in neutrophils with conflicting evidence for TLR3.^6^, ^7^, ^8^

It has been reported that asthmatic epithelial cells produce a deficient immune response in the context of a viral infection.^9^ Furthermore, airway neutrophils from asthmatics produce less inflammatory cytokines in response to lipopolysaccharide (LPS).^10^ It is currently unknown if there is dysfunction in the anti‐viral capacity of asthmatic neutrophils and whether this may contribute to viral‐induced exacerbations.

We hypothesize that neutrophils in asthmatic patients have a deficient anti‐viral response that may contribute to the pathogenesis of viral‐induced exacerbations. In this study, we aimed to determine whether neutrophils from asthmatics have an altered anti‐viral response and whether neutrophils can detect and respond to replication competent (live) RV, respiratory syncytial virus (RSV) and influenza virus infections.

## Methods

### Patient recruitment

The project protocols were approved by the Human Research Ethics Committee, The University of Sydney, prior to commencement. Patients were recruited by ethically approved flyers placed around the University of Sydney campuses and were included in the study if they were over the age of 18 and fluent in English. Exclusion criteria included pregnancy, fainting during venipuncture, having a blood‐borne infection or condition, or if asthmatics were exacerbating. All patients provided written informed consent and were asked to complete a standardized questionnaire regarding age, sex, asthma symptoms and asthma medication use. Patients also completed baseline spirometry for forced expiratory volume in 1 s (FEV_1_) and forced vital capacity (FVC) (Table 1).

**Table 1:** Patient characteristics

	Non‐asthmatic	Asthmatic
*n*	28	19
Age years, mean (± SEM)	26.11 (1.34)	34.89 (4.45)
Gender (M/F)	15/13	9/10
FEV_1_ % pred. (± SEM)	96.14 (2.13)	77.42*** (4.33)
FEV_1_/FVC % pred. (± SEM)	94.5 (3.6)	87.95* (3.04)
Short acting β_2_ agonist use (daily/weekly/less than weekly)		7/4/8
ICS use (daily/weekly/less than weekly)		10/3/6

*P < 0.05, ***P < 0.001 when compared with non‐asthmatic controls.

FEV_1_, forced expiratory volume in 1 s; FVC, forced vital capacity; ICS, inhaled corticosteroid; SEM, standard error of the mean.

### Neutrophil isolation

Neutrophils were isolated from peripheral blood collected from asthmatic and non‐asthmatic volunteers by a modified standard protocol with CD16‐positive selection.^10^ Further details are provided in Supplementary Appendix S1. HE stain was used to assess purity of neutrophils that was typically 99%. The main contaminating cell was eosinophils.

### RV16, RSV and influenza virus

RV16 was generously donated by Professor Sebastian Johnston, Imperial College, London. RV16 was grown in HeLa cells by standard procedures and infectivity titre determined by a titration assay as described.^11^

RSV (A2) was grown in Hep2a cells by standard procedures, and infectivity titre was determined by a plaque assay on Hep2a cell monolayers as described.^12^

Influenza A virus strain A/California/7/2009 (A(H1N1)pdm09) from the WHO Collaborating Centre for Reference and Research on Influenza, Melbourne, Australia, was grown in 10‐day embryonated hen's eggs by standard procedures and infectivity titre was determined by plaque assay on MDCK cell monolayers as described.^13^

### Stimulation of neutrophils with TLR agonists and respiratory viruses

Neutrophils were resuspended in 1% foetal bovine serum (Glendarach Biologicals, Melbourne, Victoria, Australia), 1% 1 M N‐[2‐hydroxyethyl]piperazine‐N′‐[2‐ethanesulphonic acid] (Life Technologies, Mulgrave, Victoria, Australia) 1% penicillin/streptomycin RPMI 1640 (Life Technologies) at 1 × 10^6^ cells/mL. Cells were unstimulated (negative control) or stimulated with EC_50_ concentrations of each TLR agonist based on dose–response curves generated for CXCL8 release (data not shown): 1 μg/mL LPS (Sigma Aldrich, Castle Hill, New South Wales, Australia), 3 μg/mL imiquimod (Invivogen, San Diego, CA, USA), 1.5 μg/mL R848 (Invivogen), 10 μg/mL poly I:C (Sigma Aldrich), except f‐met‐leu‐phe (fMLP; Sigma Aldrich) (100 nM), which was based on previous reports.^14^ Neutrophils were also stimulated with RV16 at a multiplicity of infection (MOI) of 1 infectious particle per cell as previously published.^15^ This remained consistent with RSV (MOI1) and influenza virus (MOI1). Cells were incubated at 37°C with 5% CO_2_ for 1 h NE and MMP‐9 measurements, or 24 h for CXCL8 measurements. Cell‐free supernatant and neutrophil cell pellets were collected and stored at −80°C for analysis.

### CXCL8 enzyme‐linked immunosorbent assay (ELISA)

CXCL8 production was measured using a sandwich ELISA in duplicate. Specific ELISA kits from R&D Systems (Minneapolis, MN, USA) were used according to the manufacturer's instructions. Detection limit was 15.6 pg/mL.

### NE activity assay

NE activity was measured in duplicate using a fluorescent assay from Cayman Chemicals (Ann Arbor, MI, USA) according to the manufacturer's instructions. Florescence readings from samples were compared with a standard curve of known concentrations of NE to determine the concentration. Detection limit was 3.1 ng/mL.

### MMP‐9 zymography

A bicinchoninic acid assay (Sigma Aldrich) was run for all samples according to the manufacturer's instructions to obtain the total protein concentration. Zymography was carried out according to previously published methods.^16^ Further details are provided in Supplementary Appendix S1.

### Statistical analysis

Data are represented as the mean ± standard error of the mean. Data sets with greater than 10 patients were deemed to have a normal distribution if it passed one or more of the three normality tests provided (Kolmogorov–Smirnov, D'Agostino and Pearson and Shapiro–Wilk normality tests; GraphPad Prism 5, La Jolla, CA, USA). Non‐parametric data were log_10_ transformed and normality tested again. A paired *t*‐test or one‐way analysis of variance (ANOVA) with Bonferroni post‐test was performed if the data followed a normal distribution, or a Wilcoxon matched *t*‐test or Friedman test with Dunn's multiple comparison test if data were non‐parametric. Two‐way ANOVA was performed for groups comparing asthmatic cells versus non‐asthmatic cells. Significant changes were identified where *P* < 0.05.

## Results

### Patient characteristics

The mean age of the non‐asthmatic group was 26.11 years, whereas the asthmatic patients were slightly older (34.89 years). There were approximately equal numbers of males and females in each group. The asthmatics had a significantly lower % predicted FEV_1_ and % predicted FEV_1_/FVC compared with the non‐asthmatic group and all were on a short‐acting β_2_ agonist and an inhaled corticosteroid (ICS) (Table 1).

### Bacterial compounds and viral mimics trigger the release of CXCL8 from neutrophils

We first wanted to confirm that neutrophils were able to be activated by TLR agonists that mimic viral infections and compare their response to known bacterial activators. The bacterial compounds, LPS and fMLP, induced CXCL8 with LPS inducing approximately 10‐fold more CXCL8 than fMLP. The viral surrogates (imiquimod, R848 and poly I:C) also induced release of CXCL8 from neutrophils with the TLR7/8 agonist, R848, inducing the greatest levels that was of similar magnitude to LPS stimulation (Table 2). Interleukin (IL)‐6 was not detectable after stimulation with any of the TLR agonists and, therefore, this cytokine was not quantified in subsequent experiments.

**Table 2:** CXCL8, NE and MMP‐9 release from neutrophils stimulated with bacterial compounds and viral mimics

	CXCL8 (pg/mL)	NE (ng/mL)	MMP‐9 (fold change of control)
Control	173.3 (± 22.72)	46.56 (± 5.98)	1
DMSO	241.6 (± 59.64)	49.92 (± 7)	1.23 (± 0.08)
LPS (1 μg/mL)	11 080 (± 1782)***	60.76 (± 7.08)*	2.53 (± 0.28)***
fMLP (100 nM)	1 103 (± 258.4)***	91.23 (± 6.54)***	3.44 (± 0.5)***
Imiquimod (3 μg/mL)	1 630 (± 275)***	69.92 (± 7.66)**	2.65 (± 0.42)***
R848 (1.5 μg/mL)	8 898 (± 1616)***	63.6 (± 6)*	3.76 (± 0.88)***
Poly I:C (10 μg/mL)	992.6 (± 271.5)***	52.1 (± 8.36)	1.29 (± 0.12)

CXCL8, NE and MMP‐9 release from pooled data from asthmatic and non‐asthmatic neutrophils. *P < 0.05, **P < 0.01, ***P < 0.001 compared with control conditions.

DMSO, dimethyl sulfoxide; fMLP, f‐met‐leu‐phe; LPS, lipopolysaccharide; MMP‐9, matrix metalloproteinase‐9; NE, neutrophil elastase.

### Bacterial compounds and viral mimics trigger degranulation of neutrophils

NE is the most prominent azurophilic granule product and is a measure of degranulation. fMLP and LPS increased NE release as did imiquimod and R848; however, poly I:C did not (Table 2).

MMP‐9 is predominately found in neutrophil gelatinase granules and zymography was used to detect its release by means of activity (gelatin digestion). fMLP was a potent inducer of MMP‐9 release causing approximately 3.5‐fold increase in release compared with basal levels. LPS also induced MMP‐9 but to a lesser degree. R848 was also a potent inducer of MMP‐9 release with similar levels to fMLP while imiquimod‐induced release was similar to LPS. In contrast, poly I:C did not induce MMP‐9 release from neutrophils (Table 2).

### Neutrophils derived from asthmatics had enhanced R848‐induced CXCL8 release

**Figure 1:**
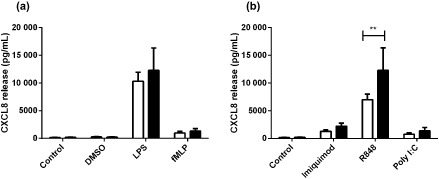
CXCL8 release sub‐analysis of neutrophils from asthmatic versus non‐asthmatic patients following stimulation with bacterial compounds and viral surrogates. CXCL8 release from asthmatic (*n* = 11 (a), *n* = 10 (b), black bars) and non‐asthmatic (*n* = 18, white bars) neutrophils stimulated with (a) bacterial compounds: lipopolysaccharide (LPS) (1 μg/mL), f‐met‐leu‐phe (fMLP) (100 nM) and dimethyl sulfoxide (DMSO) (vehicle control) and (b) viral surrogates: imiquimod (3 μg/mL), R848 (1.5 μg/mL) and polyinosinic : polycytidylic acid (poly I:C) (10 μg/mL) after 24 h. **P < 0.01 compared with non‐asthmatics.

**Figure 2:**
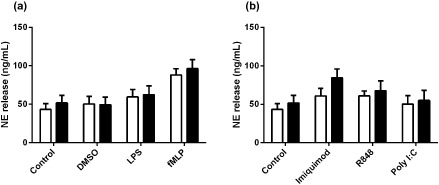
Neutrophil elastase (NE) release sub‐analysis of neutrophils from asthmatic versus non‐asthmatic patients following stimulation with bacterial compounds and viral surrogates. NE release from asthmatic (*n* = 8, black bars) and non‐asthmatic (*n* = 13, white bars) neutrophils stimulated with (a) bacterial compounds: lipopolysaccharide (LPS) (1 μg/mL), f‐met‐leu‐phe (fMLP) (100 nM) and dimethyl sulfoxide (DMSO) (vehicle control) and (b) viral surrogates: imiquimod (3 μg/mL), R848 (1.5 μg/mL) and polyinosinic : polycytidylic acid (poly I:C) (10 μg/mL) after 1 h.

**Figure 3:**
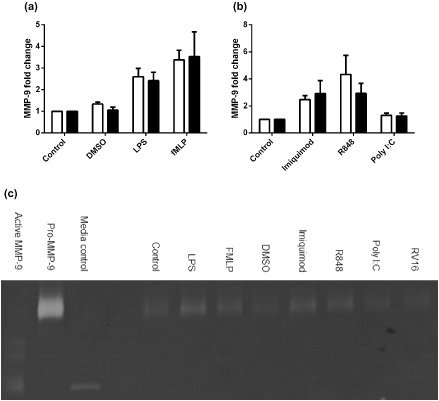
Matrix metalloproteinase (MMP)‐9 release sub‐analysis of neutrophils from asthmatic versus non‐asthmatic patients following stimulation with bacterial compounds and viral surrogates. MMP‐9 release from asthmatic (*n* = 8 (a), *n* = 9 (b), black bars) and non‐asthmatic (*n* = 13, white bars) neutrophils stimulated with (a) bacterial compounds: lipopolysaccharide (LPS) (1 μg/mL), f‐met‐leu‐phe (fMLP) (100 nM) and dimethyl sulfoxide (DMSO) (vehicle control) and (b) viral surrogates: imiquimod (3 μg/mL), R848 (1.5 μg/mL) and polyinosinic : polycytidylic acid (poly I:C) (10 μg/mL) after 1 h. (c) Representative image of a zymogram gel from one patient.

We next were interested in whether neutrophils from people with asthma had inherently altered immune responses to viral mimics that could contribute to the inflammatory milieu in the airways of these patients. We found that upon R848 stimulation (TLR7/8 agonist) neutrophils from asthmatics produced nearly twice as much CXCL8 as non‐asthmatic neutrophils (Fig. 1b). However, there were no differences found between these groups when we looked at LPS‐ and fMLP‐induced CXCL8 (Fig. 1a), NE release (Fig. 2a,b) or MMP‐9 release (Fig. 3a,b).

### Respiratory viruses differentially modulate neutrophil responses

**Figure 4:**
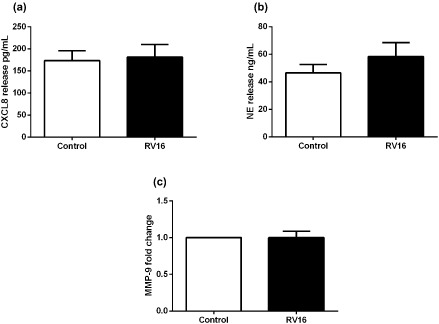
CXCL8 levels and degranulation products of neutrophils stimulated with RV16. (a) CXCL8, (b) neutrophil elastase (NE) and (c) matrix metalloproteinase (MMP)‐9 release from pooled asthmatic and non‐asthmatic neutrophils stimulated with RV16. *n* = 29 (a), *n* = 21 (b), *n* = 22 (c).

As neutrophils released CXCL8, NE and MMP‐9 in response to TLR agonists, we next determined the ability of infectious RV16 to induce these mediators from neutrophils. RV was chosen due to the high prevalence in the community and did not induce CXCL8, NE or MMP‐9 release from neutrophils (Fig. 4a–c).

**Figure 5:**
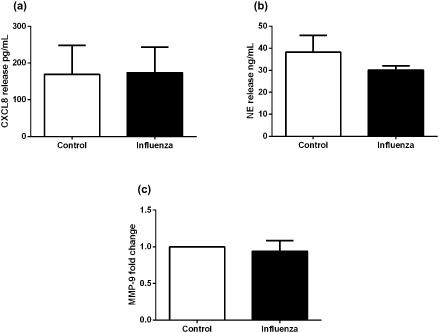
CXCL8 levels and degranulation products of neutrophils stimulated with influenza virus. (a) CXCL8, (b) neutrophil elastase (NE) and (c) matrix metalloproteinase (MMP)‐9 release from pooled asthmatic and non‐asthmatic neutrophils stimulated with influenza virus. *n* = 6, except (a) where *n* = 5.

Influenza virus also has been detected in the airways of exacerbating asthmatics.^17^ Neutrophils stimulated with influenza virus also did not induce CXCL8, NE or MMP‐9 (Fig. 5a–c).

**Figure 6:**
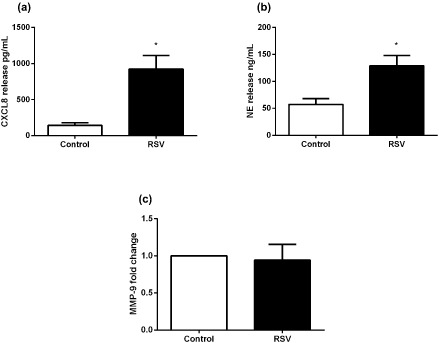
CXCL8 levels and degranulation products of neutrophils stimulated with RSV. (a) CXCL8, (b) neutrophil elastase (NE) release and (c) matrix metalloproteinase (MMP)‐9 release from pooled asthmatic and non‐asthmatic neutrophils stimulated with RSV. *n* = 5, except (a) where *n* = 4. *P < 0.05 compared with control.

We next investigated RSV as it is another common respiratory virus detected in the airways of exacerbating asthmatics, particularly in children.^17^ Unlike RV16 or influenza virus, RSV did induce CXCL8 (Fig. 6a) and NE (Fig. 6b); however, MMP‐9 was not detected (Fig. 6c).

We also assessed viral interaction with neutrophils by detecting viral RNA in a subset of neutrophil cell pellets by reverse transcription polymerase chain reaction. We found that RV and RSV RNA were present in neutrophil cell pellets 24 h post‐stimulation (Supplementary Figs S1 & S2). We were unable to detect influenza in our samples due to technical limitations.

## Discussion

This is the first study to comprehensively explore classical neutrophil functions utilized against bacteria in the context of viral infections. We compared neutrophil responses against simulated bacterial and viral infections, immune responses between asthmatic and non‐asthmatic neutrophils and finally neutrophil responses between viral surrogates and live viruses. We found that neutrophils were able to respond, by means of CXCL8, NE and MMP‐9 release, to a variety of TLR agonists that mimic viral infections.

Notably, asthmatic neutrophils produced significantly more CXCL8 in response to R848 that has not been previously demonstrated. R848 non‐specifically binds to both TLR7 and 8 and as no difference was observed with the specific TLR7 agonist, imiquimod, it can be concluded that the observation is due to dysregulated TLR8 activation. This may have substantial implications as the expression of TLR8 in neutrophils is the greatest of the three TLRs investigated, and TLR8 is not expressed in eosinophils.^6^, ^7^ Oxidative stress has been confirmed in asthmatic airways by analysis of bronchial alveolar lavage fluid^18^ and has been reported to augment TLR8 activation,^19^ which may potentially exacerbate neutrophilic inflammation. Genetic studies have also linked TLR7 and TLR8 polymorphisms to increased risk of asthma.^20^

Interestingly, out of the three respiratory viruses tested, only RSV was able to activate neutrophils even though TLR ligands could cause release of all mediators investigated. Neutrophils, in principle, are unable to differentiate between different types of viruses; rather they recognize PAMPs via TLRs. The majority of respiratory viruses are single‐stranded RNA viruses that are detected by TLR3,7 and 8 of which neutrophils predominately express TLR8, low levels of TLR7 and little to no TLR3.^6^, ^7^, ^8^ We speculate that the inability of RV and influenza to replicate in neutrophils could explain our results as the limited amount of viral RNA derived from the initial infection may be insufficient to activate the TLRs. Furthermore, RSV has a limited capacity to replicate within neutrophils that agrees with this explanation.^21^

Our study suggests that direct activation of neutrophils by virus may not be the main mechanism involved in these infections, particularly RV infections. Many respiratory viruses primarily infect the epithelium and the resulting immune response may initiate a robust neutrophilic response. Other immune cells may also be activated and could potentially cross‐talk with neutrophils to activate them, which could be an area of further investigation. As live viruses, in general, had a limited capacity to activate neutrophils, we did not have the statistical power required for sub‐analysis between asthmatic and non‐asthmatic neutrophils stimulated with respiratory viruses. We speculate that differential responses to RSV might occur given our observation with R848.

Neutrophils are associated with a variety of lung diseases including asthma and are believed to be dysfunctional in disease states. Baines *et al*. demonstrated that airway neutrophils had impaired CXCL8, IL‐1β and tumour necrosis factor α release in response to LPS; however, blood neutrophils did not, albeit there was a similar trend.^10^ Similarly, we also found that blood neutrophils did not have impairment of the LPS response in respect to CXCL8 release. This demonstrates the potential differences between sputum and blood neutrophils. Alternatively, the differences between this and the previous study may be due to age differences between the asthmatics (35 in this study versus 59) as neutrophil function changes with age,^22^ or the severity of their asthma as we recruited patients who had milder asthma in comparison with the previously published study.

In this study, we were limited to the use of peripheral blood neutrophils and despite their limitation they still provide valuable insights into the pathophysiology of asthma. Neutrophils are short‐lived inflammatory cells (12–24 h), and during viral infections neutrophilia occurs as a result of neutrophil migration from the blood. Therefore, we believe that studying blood neutrophils is an appropriate model to use for investigating the effects of acute viral infections.

Viral surrogates have been extensively used by our group to mimic viral infections as well as to study TLR activation.^23^ We demonstrated that neutrophils have the relevant receptors to become activated and release CXCL8 in the context of both bacterial and viral infections. CXCL8 is a potent chemoattractant for neutrophils which is released by a variety of cells, including neutrophils,^24^ which can therefore amplify recruitment in a positive feedback manner if not tightly regulated. Interestingly, CXCL8 could not be detected in supernatants 1 h post‐stimulation, displaying *de novo* synthesis of this cytokine. All the stimulants used were able to induce CXCL8 release, albeit to varying degrees. It may be tempting to relate these differing responses to receptor expression levels but different potencies of our agonists, being full or partial agonists, or intrinsic differences in downstream signalling cascades could explain our observations; however, exploration of this mechanism was beyond the scope of this study.

Degranulation is an important effector function of neutrophils. NE and MMP‐9 are two of many components found in granules and are particularly important in clearing Gram‐negative bacteria and migration, respectively.^25^, ^26^ All stimulants, except poly I:C, induced release of NE and MMP‐9. Poly I:C also had low potency in CXCL8 release. TLR3, the receptor for poly I:C, is believed to not be present in neutrophils that would partly explain our observations.^7^ Poly I:C may also be a ligand for other intracellular RNA receptors such as melanoma differentiation‐associated protein 5 (MDA‐5),^27^ and induction of CXCL8 may be due to MDA‐5 activation rather than TLR3.

The patients in this study were predominately mild asthmatics who were regularly taking ICS. Inhaled steroid use is potentially a confounding and limiting factor of this study as steroids increase the lifespan of neutrophils by inhibiting apoptosis^28^ If steroids were effecting neutrophil function, it may be reasonable to assume that all outputs would be different; however, we only observed greater responses with the TLR8 agonist. As this study mainly consisted of mild asthmatics, it would be interesting to investigate if differences would be seen in neutrophils from a more severe cohort.

In conclusion, we have found that neutrophils contain the relevant components to detect and respond to both bacteria and viruses. Of importance, was our finding that TLR8 activation resulted in greater CXCL8 production from asthmatic neutrophils, which may contribute to neutrophilic inflammation in the airways. Our data suggest that neutrophils are unlikely to be directly activated by RV during asthma exacerbations. We propose that airway epithelium or smooth muscle, both of which are known to be significantly modulated in the context of asthma,^29^, ^30^ may play an important role in initiating a cascade of events during infection that results in extensive neutrophilic inflammation. Alternatively, it is not activation of neutrophils that is significant during exacerbations, rather the resolution or regulation of neutrophilic inflammation following infection that may be the determining factor in exacerbations. Animal models of lung viral infection clearly show that inhibition of lung neutrophilia causes detrimental outcomes,^31^, ^32^, ^33^ and defective resolution of inflammation is hypothesized to contribute to the chronicity of the disease.^34^ The question now posed is; are asthma exacerbations prolonged by excessive neutrophilic inflammation?

## Acknowledgements

This study was funded by the National Health and Medical Research Council (NHMRC), Australia. J.K.B. was supported by a NHMRC Career Development Fellowship #1032695. B.G.O. was supported by a NHMRC Career Development Fellowship #1026880.

## Supporting information


**Figure S1** Detection of RV16 RNA by reverse transcription (RT)‐PCR in neutrophil pellets 24 h post‐stimulation with RV16 (MOI1).
**Figure S2** Detection of RSV RNA by reverse transcription (RT)‐PCR in neutrophil pellets 24 h post‐stimulation with RSV (MOI1).
**Appendix S1** Methods.
