# Supplementary material for: Differential neutrophil activation in viral infections: Enhanced TLR‐7/8‐mediated CXCL8 release in asthma
Source: Respirology. 2015 Oct 18;21(1):172–9. doi: 10.1111/resp.12657 (PMC5324549; doi:10.1111/resp.12657)
Supplement: Supplementary file 1 — Figure S1 Detection of RV16 RNA by reverse transcription (RT)‐PCR in neutrophil pellets 24 h post‐stimulation with RV16 (MOI1). Figure S2 Detection of RSV RNA by reverse transcription (RT)‐PCR in neutrophil pellets 24 h post‐stimulation with RSV (MOI1). Appendix S1 Methods. [file RESP-21-172-s001.docx]

**SUPPLEMENTARY INFORMATION**

**Differential neutrophil activation in viral infections: Enhanced TLR-7/8-mediated CXCL8 release in asthma**

Francesca S.M. Tang ^1,2^, David Van Ly^1,3^, Kirsten Spann^4^, Patrick C. Reading^5^, Janette K. Burgess^1,2^, Dominik Hartl^6^, Katherine J. Baines^7^ and Brian G. Oliver^1,8^

^1^Woolcock Institute of Medical Research, The University of Sydney , NSW 2006 Australia

^2^Discipline of Pharmacology, School of Medical Sciences, Faculty of Medicine, The University of Sydney , NSW 2006 Australia

^3^Genome Integrity Group, The Children's Medical Research Institute, NSW 2145 Australia

^4^School of Biomedical Science, Faculty of Health, Queensland University of Technology, QLD 4059 Australia

^5^WHO Collaborating Centre for Reference and Research on Influenza, Peter Doherty Institute for Infection and Immunity, 792 Elizabeth St, Melbourne, VIC 3000 Australia

^6^Department of Pediatrics I, University of Tübingen, Tübingen 72076 Germany

^7^Priority Research Centre for Asthma and Respiratory Disease, The University of Newcastle, NSW 2308 Australia

^8^School of Medical and Molecular Biosciences , University of Technology Sydney , NSW 2007 Australia

**APPENDIX 1-**METHODS

**Neutrophil isolation from peripheral blood**

Blood was mixed with acid citrate dextrose (ACD), 10mL of phosphate buffered saline (PBS) (Life Technologies) and 6mL of 10% dextran (MP Biomedicals, Santa Ana, USA) and left for 20 minutes for sedimentation to occur at room temperature. The top layer was removed, overlayed on Ficoll Paque-PLUS (GE Healthcare, Rydalmere, Australia) and centrifuged at 490g for 10 minutes. The supernatant was discarded and the cell pellet of granulocytes was resuspended in sterile water for 30 seconds to lyse red blood cells (RBCs) before osmolarity was re-established with equal parts of 2x PBS. Cells were then incubated for 30 minutes at 4°C with CD16 magnetic beads (Miltenyi Biotec, North Ryde, Australia) before running through a magnetic column as per the manufacturer’s instructions.

**IL-6 enzyme-linked immunosorbent assay (ELISA)**

IL-6 was measured using a sandwich ELISA in duplicate. Antibodies against IL-6 from BD Bioscience (Franklin Lakes, USA) were used according to the manufacturer’s instructions. Detection limit was 7.8 pg/mL.

**MMP-9 zymography**

200ng of total protein was loaded into each lane of a 1% gelatin polyacrylamide gel. The gel was run and then proteinases were activated in a CaCl_2_ activation buffer overnight before staining with Coomassie brilliant blue dye. Bands were determined to be pro-MMP-9 using size markers and MMP-9 standards. Densitometry was performed with Carestream Molecular Imaging Software on images taken by a Kodak Image station from Integrated Sciences (Chatswood, Australia) to determine the relative fold change compared to control. Control samples for each patient were loaded on each gel to normalise across different gels, if required.

**Real-time polymerase chain reaction (RT-PCR)**

Control and virus stimulated cell pellets from the 24 hour time point were lysed and mRNA collected using the Nucleospin RNA kit (Machery Nagel, Düren, Germany) as per the manufacturer’s instructions. mRNA was converted into cDNA using M-MLV reverse transcriptase (Life Technologies) and PCR run using Biotaq DNA polymerase system (Bioline, Alexandria, Australia) with targets for RV16 and RSV-A according to the manufacturer’s instructions. Water was used as a negative control and pure virus RNA was extracted and loaded as the positive control. Annealing temperature of 60°C and 35 cycles were used.

**Figure S1**


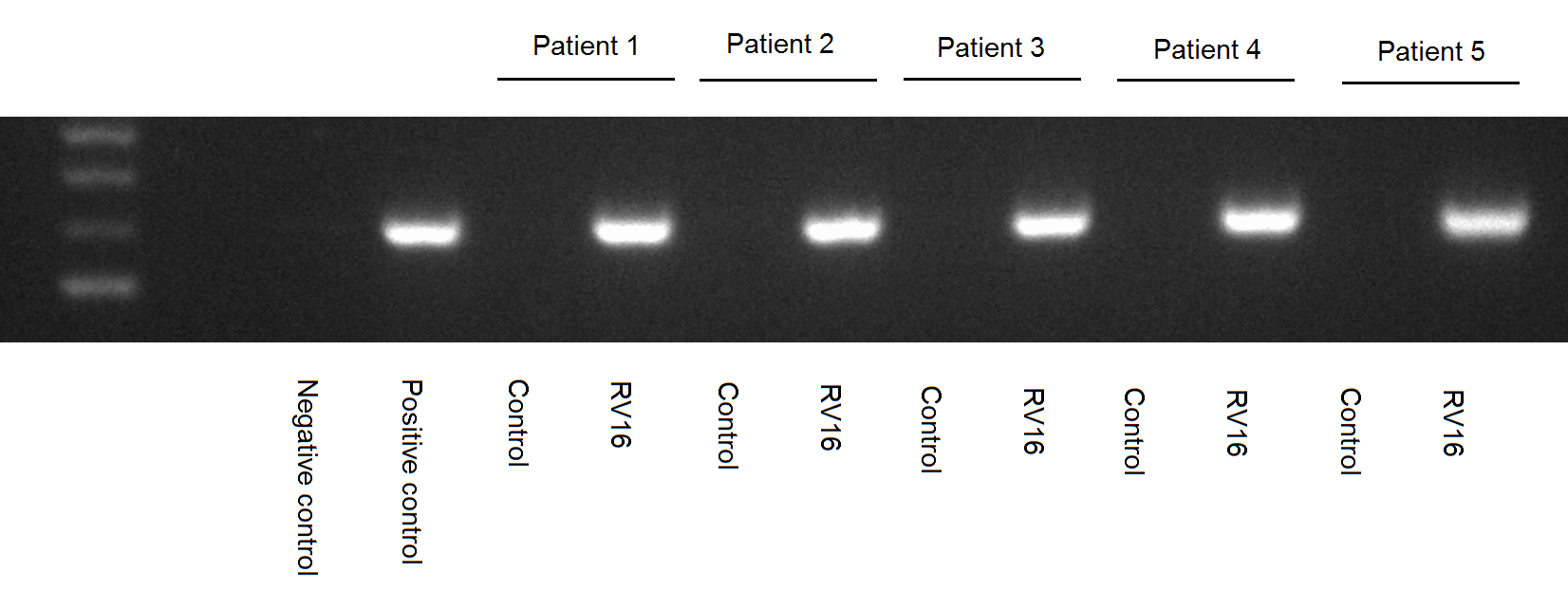


Detection of RV16 RNA by reverse transcription (RT)-PCR in neutrophil pellets 24 hours post-stimulation with RV16 (MOI1).

**Figure S2**


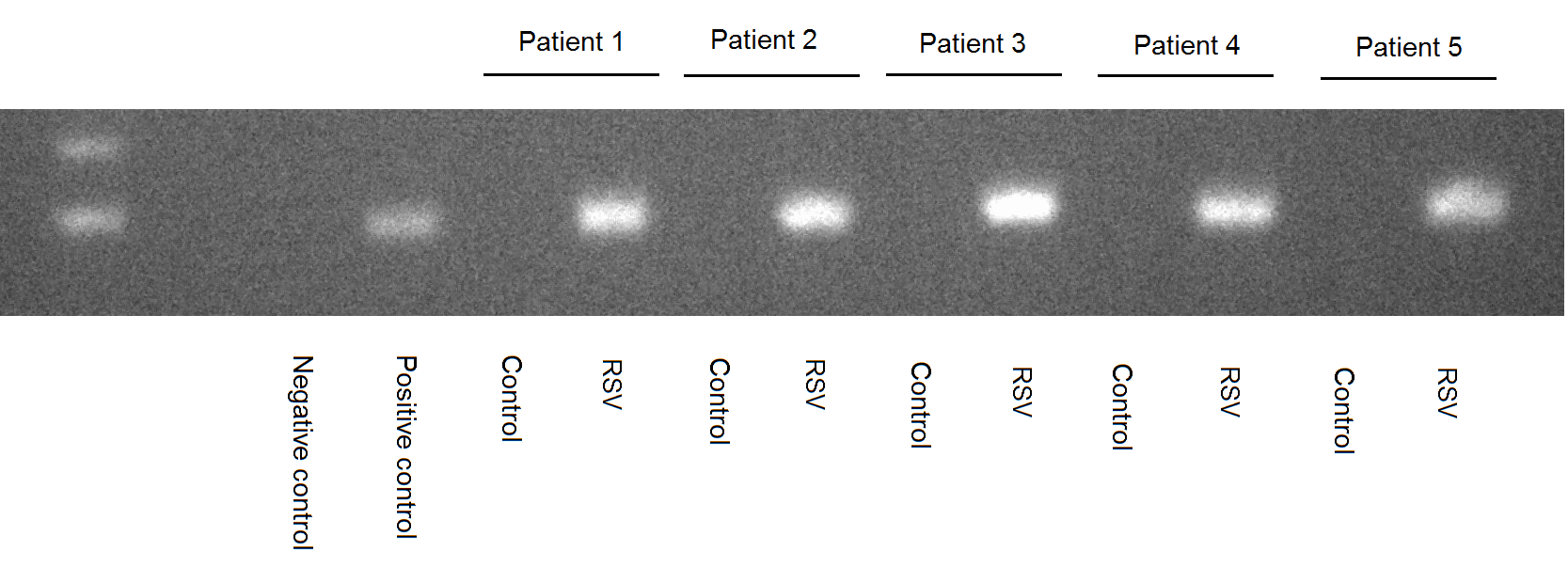


Detection of RSV RNA by reverse transcription (RT)-PCR in neutrophil pellets 24 hours post-stimulation with RSV (MOI1).
